# Association of chronic diabetes and hypertension in sural nerve morphometry: an experimental study

**DOI:** 10.1186/s13098-015-0005-8

**Published:** 2015-02-15

**Authors:** Luciana Sayuri Sanada, Marcelo Rodrigo Tavares, Karina Laurenti Sato, Renata da Silva Ferreira, Milena Cardoso Maia Neubern, Jaci Ayrton Castania, Helio Cesar Salgado, Valéria Paula Sassoli Fazan

**Affiliations:** Department of Neuroscience and Behavioral Neurosciences, School of Medicine of Ribeirão Preto, University of São Paulo, Ribeirão Preto, SP Brazil; Department of Anatomy, José do Rosário Velano University, Alfenas, MG Brazil; Departament of Physical Therapy, Federal University of Sergipe, Aracaju, SE Brazil; Department of Physiology, School of Medicine of Ribeirão Preto, University of São Paulo, Ribeirão Preto, SP Brazil; Department of Surgery and Anatomy, School of Medicine of Ribeirão Preto, USP, Av. Bandeirantes 3900, Ribeirão Preto, SP Brazil

**Keywords:** Sural nerve, Myelinated fibers, Morphometry, Experimental hypertension, Experimental diabetes, Spontaneously hypertensive rats, Streptozotocin

## Abstract

**Background:**

Prospective studies have shown incidence rates of hypertension in diabetes mellitus to be three times that of subjects without diabetes mellitus. The reverse also applies, with the incidence of diabetes two to three times higher in patients with hypertension. Despite this common clinical association, the contribution of each isolated entity in the development of a neuropathy is still not well understood. The aims of the present study were to investigate the presence of peripheral neuropathy in spontaneously hypertensive rats (SHR) and SHR with chronically induced diabetes, using a morphological and morphometric study of the sural nerves.

**Methods:**

Female SHR and normotensive Wistar rats (WR), 8 weeks old, received a single intravenous injection of streptozotocin (STZ) through the tail vein. Controls from both strains received vehicle. Twelve weeks after the injection, sural nerves were dissected and prepared for light microscopy. Morphometry of sural nerve fascicles and myelinated fibers was performed with the aid of computer software.

**Results:**

The sural nerve myelinated fibers were highly affected by experimental diabetes in normotensive rats, causing mainly the reduction of the fiber size. Hypertensive rats showed characteristics of small fiber neuropathy and a severe reduction of the number and density or Schwann cells. The association between diabetes and hypertension caused an increase on the average size of the myelinated fibers, pointing to a small fiber loss, associated to axonal atrophy.

**Conclusions:**

Our study gives morphological support to the existence of a neuropathy due to hypertension, which is among one of the most common risk factors for diabetic neuropathy. The association between the two neuropathies showed to be a complex alteration, involving and including both, large and small fibers neuropathy. Hypertension caused, indeed, an exacerbation of the alterations already observed in experimental models of diabetic neuropathy.

## Background

It has been pointed out that hypertension is a main risk factor for stroke and vascular dementia [1]. The important changes to the cerebrovascular tree caused by hypertension turn the brain more susceptible to infarcts, microaneurysms and ischemia [1]. On the peripheral nervous system, alterations in endoneural blood vessels can cause morphological and morphometric changes in peripheral nerves [1-4]. Previous results from our laboratory [4] showed that the sustained high blood pressure in adult spontaneously hypertensive rats (SHR) affected the sural nerve myelinated fibers, morphologically and morphometrically.

Spontaneously hypertensive rats (SHR), first inbred from Wistar-Kyoto rats (WKY), are considered a good experimental model of human essential hypertension [5,6], and it is expected that WKY would be used as the normotensive controls of SHR in diverse experimental protocols. Nevertheless, Wistar rats (WR) have been recently used as the SHR controls as often as the WKY [7], mainly because WKY are not readily or easily available. Sanada et al. [7] showed that sural nerve morphology is similar between WKY and WR, allowing the use of WR as the SHR controls in morphological investigations involving peripheral neuropathies.

Prospective studies have shown incidence rates of hypertension in diabetes mellitus to be three times that of subjects without diabetes mellitus. The reverse also applies, with the incidence of diabetes two to three times higher in patients with hypertension [8,9]. Tesfave et al. [10] found an association between the prevalence of diabetic neuropathy and the presence of cardiovascular disease, as hypertension. Despite this common clinical association, the contribution of each isolated entity in the development of a neuropathy is still not well understood and few reports deal with the experimental diabetes associated to hypertension in morphological and/or morphometric alterations of the peripheral nerves of rats.

We aimed to investigate the possible alterations of morphological and morphometric parameters of sural nerves fascicles and myelinated fibers, in adult female rats, with well-established hypertension. We also compared the effects on nerve morphology of chronic induced diabetes associated with hypertension.

## Materials and methods

All procedures adhered to “The ARRIVE guidelines: Animal Research: Reporting In Vivo Experiments, originally published in PLoS Biology, June 2010” and were approved by the Institutional Ethics Committee for Animal Research (CETEA - Comitê de Ética em Experimentacão Animal, protocol number 030/2006). A conscious effort was done to minimize the number of animals used.

Experiments were performed on female SHR and Wistar rats, born and raised in a carefully regulated environment maintained at 21°C - 23°C, 40% - 70% relative air humidity, and 12/12 hr light/dark cycle, receiving tap water and normal rat chow ad libitum, throughout the experiment. Four experimental groups were used (N = 6 for each group): 1) Wistar control (WR) - animals which received only vehicle (citrate buffer) 12 weeks before the experiments; 2) Wistar Diabetic (WR + STZ) - rats which received streptozotocin (STZ) injection 12 weeks before the experiments; 3) SHR control (SHR) - animals which received only vehicle (citrate buffer) 12 weeks before the experiments; 4) SHR Diabetic (SHR + STZ) - rats which received STZ injection 12 weeks before the experiments. Experimental diabetes was induced in female rats with 8 weeks of age through STZ (60 mg/kg) intravenous injection as described previously [11-14]. The vein used for the injections, either of STZ or vehicle was the tail vein. The animals were considered diabetic when the blood glucose levels were higher than 350 mg/dl. On the STZ injected animals, the onset of diabetes occurred rapidly and was identified by polydipsia and polyuria. Non-fasting blood glucose (mg/dl) was determined with a glucose analyzer (Beckman Instruments, Inc., Brea, CA, USA) 3 days after STZ injection and immediately before the experiments, in blood droplets collected from an incision at the tip of the tail.

On the final experimental day (12 weeks after injections), animals were anesthetized with sodic thiopental (Thionembutal, 40 mg kg, i.p.) and a catheter was inserted into the femoral artery for measurement of arterial pressure (AP). Recordings of the systolic (SAP), diastolic (DAP), mean arterial pressure (MAP) and heart rate (HR) were performed as described elsewhere [15,16]. After the recordings, rats were perfused through the left ventricle first with a 0.05 M phosphate-buffered saline solution, pH 7.4 and then with a 2.5% glutaraldehyde solution in 0.1 M cacodylate buffer, pH 7.2. Both right and left sural nerves, from their origin in the hip (5–7 mm distal to the greater trochanter) through their distal branching at the lateral malleolus level, were carefully dissected without stretching, removed in one piece and placed in the fixative solution for an additional 12 hour. They were washed in cacodylate buffer, pH 7.2, and proximal (close to the origin) and distal (close to terminal branching) segments (of approximately 3 mm each) were cut and processed for epoxy resin embedding (EMbed-812®, Electron Microscopy Sciences, Hatfield, PA, USA) as described previously [2,7,17,18]. Samples of all four experimental groups were histologically processed at once so that they were submitted to absolutely the same experimental conditions throughout the experiments.

Semithin (0.5 μm thick) transverse sections of the fascicles were stained with 1% toluidine blue and examined with the aid of an Axiophot II photomicroscope (Carl Zeiss, Jena, Germany). The images were sent via a digital camera (TK- 1270, JVC, Victor Company of Japan Ltd, Tokyo, Japan) to an IBM/PC where they were digitized. The study of nerve fascicles, myelinated fibers and endoneural space were performed following the methods developed in our laboratory [2,7,17-21]. Morphometric data obtained were: fascicular area and diameter, myelinated fiber number and density, Schwann cell nuclei number and density, area and diameter of each myelinated fiber (defined by the axon and its respective myelin sheath, excluding the Schwann cell nucleus when present) and respective axon (fiber excluding the myelin sheath) and myelin sheath area of each fiber present in the endoneural space and the g ratio (the ratio between the axon diameter and total fiber diameter - which indicates the degree of myelination) [22,23]. The percentage of the total cross-sectional area of the endoneural space occupied by the blood vessels was calculated, and hereafter referred as the capillary of occupancy. The computer software used for the morphometry process was the KS 400, Kontron 2.0 (Eching Bei München, Germany).

Morphometric data were tested for normal distribution by the Kolmogorov-Smirnov normality test followed by the Levene test for variance equivalence. If data presented a normal distribution and equivalent variance, comparisons were made between proximal and distal segments in the same group by paired Student’s t-test. Otherwise, comparisons were made by Wilcoxon’s nonparametric test for paired samples. For comparisons between right and left segments in the same group, normally distributed data were tested using the unpaired Student’s t-test. Alternatively comparisons were made by the Mann–Whitney non-parametric test. Comparisons between groups either for physiological or morphometric data were made by one-way analysis of variance (ANOVA) followed by Holm-Sidak post hoc test. Comparisons between histograms were made by one-way analysis of variance (ANOVA) on Ranks provide that the distributions did not pass the normality test. For all applied statistical tests, differences were considered significant when p < 0.05. Data are presented as mean ± standard error of the mean (SEM).

## Results

Body weight, blood glucose level, MAP and HR values for all experimental groups 12 weeks after the injections are shown in Table 1. Hyperglycemia observed after 12 weeks of STZ injection reached similar levels in WR and SHR rats. There was a continuous body weight gain over time since the injections in animals from all experimental groups but STZ-injected animals did not gain as much weight as their respective controls.

**Table 1:** **Body weight, blood glucose level, mean arterial pressure (MAP) and heart rate (HR) data, at 12 weeks after injections, from normotensive Wistar rat control group (WR), normotensive Wistar rat with chronic induced diabetes group (WR + STZ), spontaneously hypertensive rat group (SHR), spontaneously hypertensive rat with chronic induced diabetes group (SHR + STZ)**

	**Body weight (g)**	**Blood glucose (mg/dl)**	**MAP (mmHg)**	**HR (bpm)**
WR	363 ± 8	97 ± 6	119 ± 13	312 ± 46
WR + STZ	243 ± 25	355 ± 38^*#^	116 ± 6^#^	269 ± 31
SHR	193 ± 6^*^	96 ± 1	180 ± 7	310 ± 12
SHR + STZ	153 ± 9^*^	305 ± 35^*#^	181 ± 11	373 ± 12^+^

*indicates significant difference compared to Normotensive Wistar rat (WR) group; + indicates significant difference compared to Normotensive Wistar rat with chronic induced diabetes (WR + STZ) group; # indicates significant difference compared to Spontaneously hypertensive rat (SHR) group.

**Figure 1:**
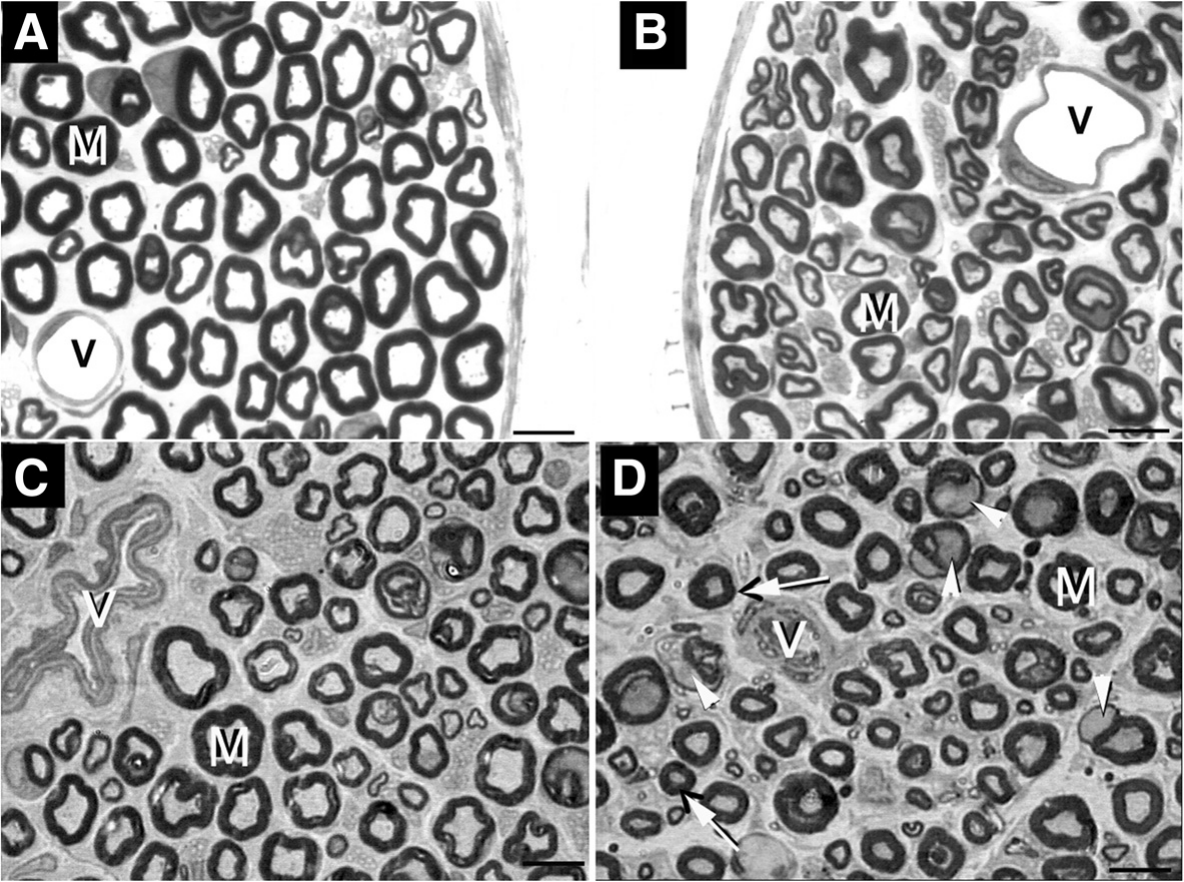
**Representative semithin cross section of the sural nerve from normotensive Wistar rat. (A)**, normotensive Wistar rat with chronically induced diabetes **(B)**, Spontaneously hypertensive rat **(C)** and Spontaneously hypertensive rat with chronically induced diabetes **(D)**, showing typical endoneural structures. Large (M) and small myelinated fibers are present in the endoneural space. Note the presence of normal endoneural vessels (V) in A and B while in C and D, the vessels show thickening of the walls and reduced lumen. In D, arrows indicate axons with atrophy and arrowheads indicate myelinated fibers with severe myelin disruption. Toluidine blue stained. Bar = 10 μm.

All sural nerves included in this study showed good preservation of structures and general morphological characteristics of the sural nerve fascicles of WR, WR + STZ, SHR and SHR + STZ were similar to those previously described [2,7,17,18]. The comparisons between segments for all groups showed no endoneural morphological differences on the same side. Likewise, comparison between nerves from right and left sides showed no differences for all groups. However, the comparison between strains indicated that the sural nerves from SHR and SHR + STZ showed a larger number of collapsed blood vessels and/or vessels with thickening of the wall (Figure 1). In addition, some myelinated fibers with degenerative signs were present in both SHR groups. Moreover, in SHR + STZ group a larger number of myelinated fibers with smaller caliber and thickening of the myelin sheath could be observed (Figure 1).

The statistical analysis of the morphometric data, for fascicles and fibers, did not show intra-group differences, neither between segments (proximal and distal on the same side), nor between sides. Thus, morphometric results will be presented and discussed based on the comparison between the different experimental groups.

Fascicle morphometric data are shown in Table 2. The fascicular area of sural nerve in SHR + STZ was significantly smaller compared to WR and WR + STZ groups (p < 0.001). The same observation was made for the myelinated fiber number, Schwann cell nuclei number, Schwann cell nuclei density and capillary percentage of occupancy (p < 0.001). Myelinated fiber number in SHR group was significantly smaller compared to WR and WR + STZ groups. On the other hand, SHR + STZ and SHR groups showed a higher myelinated fiber density compared to WR and WR + STZ groups (p < 0.001).

**Table 2:** **Average morphometric data of sural nerves fascicles from the four experimental groups: normotensive Wistar rat control group (WR), normotensive Wistar rat chronically induced diabetes group (WR + STZ), spontaneously hypertensive rat group (SHR), spontaneously hypertensive rat chronically induced diabetes group (SHR + STZ)**

	**WR**	**WR + STZ**	**SHR**	**SHR + STZ**
Fascicular area (μm^2^)	76963 ± 4361	64739 ± 3732	50018 ± 2047	42670 ± 2345^*+^
Fascicular diameter (μm)	202 ± 24	169 ± 17	153 ± 10	156 ± 11
Myelinated fiber number	1079 ± 82	1039 ± 50	849 ± 28^*+^	767 ± 28^*+^
Myelinated fiber density (number of fibers/μm^2^)	13994 ± 601	16894 ± 1113	17206 ± 391^*+^	18471 ± 684^*+^
Schwann cell nucleus number	60 ± 5	66 ± 4	51 ± 2	28 ± 2^*+#^
Schwann cell nucleus density (number of nucleus/μm^2^)	789 ± 60	1094 ± 103	1055 ± 60*^+^	670 ± 77^+#^
Capillary occupancy (%)	0.90 ± 0.18	0.91 ± 0.17	0.43 ± 0.08	0.27 ± 0.06^*+^

*indicates significant difference compared to Normotensive Wistar rat (WR) group; + indicates significant difference compared to Normotensive Wistar rat with chronic induced diabetes (WR + STZ) group; # indicates significant difference compared to Spontaneously hypertensive rat (SHR) group.

Myelinated fiber morphometric data are shown in Table 3. The control group (WR) showed significantly higher values of myelinated fiber area and diameter compared to all other experimental groups (p < 0.001). Similar results were observed for the myelin sheath area (p < 0.001) but this parameter was also significantly higher in WR + STZ group when compared to SHR and SHR + STZ groups. Myelinated axon area was significantly smaller in WR + STZ group when compared to SHR and SHR + STZ group. There was a tendency towards larger myelinated axon diameter on both SHR groups, and this was reflected on the g ratio values, being the SHR g ratio average larger than both WR groups.

**Table 3:** **Average morphometric data of sural nerves myelinated fibers from the four experimental groups: normotensive Wistar rat control group (WR), normotensive Wistar rat chronically induced diabetes group (WR + STZ), spontaneously hypertensive rat group (SHR), spontaneously hypertensive rat chronically induced diabetes group (SHR + STZ)**

	**WR**	**WR + STZ**	**SHR**	**SHR + STZ**
Myelinated fiber area (μm^2^)	33.46 ± 0.74	29.00 ± 0.89*	25.65 ± 0.62*	27.47 ± 1.47*
Myelinated fiber diameter (μm)	5.48 ± 0.07	5.10 ± 0.08*	4.78 ± 0.07*	4.81 ± 0.10*
Myelin sheath area (μm^2^)	25.69 ± 0.55	21.56 ± 0.80*	16.49 ± 0.40*^+^	17.90 ± 0.75*^+^
Myelinated axon area (μm^2^)	8.19 ± 0.25	7.38 ± 0.26	9.16 ± 0.25^+^	9.56 ± 0.54^+^
Myelinated axon diameter (μm)	2.67 ± 0.05	2.65 ± 0.05	2.86 ± 0.04	2.80 ± 0.09
g ratio	0.49 ± 0.01	0.53 ± 0.01	0.61 ± 0.01*^+^	0.60 ± 0.01

*indicates significant difference compared to Normotensive Wistar rat (WR) group; + indicates significant difference compared to Normotensive Wistar rat with chronic induced diabetes (WR + STZ) group.

**Figure 2:**
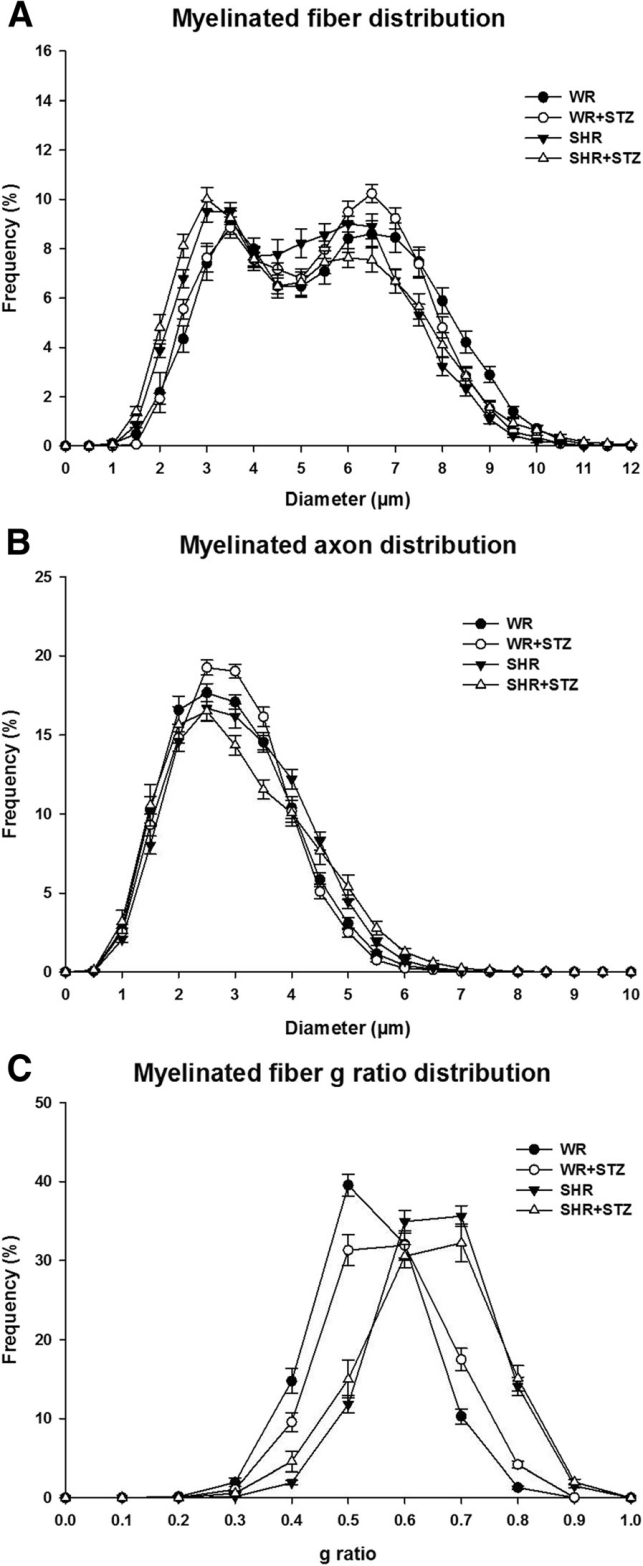
**Size distribution histograms of myelinated fibers. (A)**, respective axons **(B)** and g ratio **(C)** of sural nerve in normotensive Wistar rat (WR), normotensive Wistar rat with chronically induced diabetes (WR + STZ), Spontaneously hypertensive rat (SHR) and Spontaneously hypertensive rat with chronically induced diabetes (SHR + STZ). WR + STZ showed a higher peak at larger fibers while the SHR + STZ showed a lower peak of these fibers compared to all other groups. Myelinated axons presented a slight shift to smaller size in SHR + STZ group compared to all others. The g ratio distributions in both SHR groups are shifted to the right compared to normotensive groups.

Distributions of myelinated fiber and axon diameters and g ratio values are shown in Figure 2. Myelinated fiber diameters ranged from 1.0 to 11.0 μm in all four groups, with a bimodal distribution. For the SHR groups, the small myelinated fiber peak was at 2.5 μm while both WR presented peaks at 3.5 μm. For the large myelinated fibers, all four groups presented peaks at 6.5 μm but the WR + STZ showed a larger percentage of these fibers while the SHR + STZ showed a smaller percentage of these fibers compared to all other groups. No differences between the large fibers peak was observed between WR and SHR. The myelinated fiber axon diameters ranged from 0.5 to 6.5 μm in all groups distributed in an unimodal shape, with peak at 2.5 μm in all groups, with no differences between groups.

For the g ratio distributions, the range was between 0.2 and 1.0 for all groups but with significant differences on the peaks between groups. While WR presented peak at 0.5, WR + STZ showed a plateau between 0.5 and 0.6. For both SHR groups, there was a plateau between 0.6 and 0.7 with higher percentage of fibers with these values on SHR groups, compared to the SHR + STZ.

## Discussion

For a long time, our laboratory has been dedicated to investigate abnormalities in the morphology and morphometry of peripheral nerves due to hypertension [2,3,7,24-27], pointing to the description of a so called “hypertensive neuropathy”. In fact, it was recently described that non-diabetic patients with chronic untreated hypertension may develop impairment of nerve function [28]. The positive correlation between hypertension and diabetes described in humans drove us to investigate this association under controlled experimental conditions using SHR and STZ-induced diabetes. Our study showed that the sural nerve myelinated fibers were highly affected by experimental diabetes, causing mainly the reduction of the fiber size. But the most striking result from this work is that the association between diabetes and hypertension caused an increase on the average size of the myelinated fibers, pointing to a small fiber loss, associated to axonal atrophy.

Abnormalities reported in human diabetic neuropathy include axonal degeneration, primary demyelination resulting from Schwann cell dysfunction, secondary segmental demyelination related to impairment of the axonal control of myelination, remyelination, proliferation of Schwann cells, atrophy of denervated bands of Schwann cells, onion-bulb formations and hypertrophy of the basal lamina [29,30]. Animal models of diabetes still do not reproduce all morphological alterations present in human nerves, but offer the possibility of investigating the disease under controlled risk factors environment, including the association with co-morbidities or risk factor for the disease.

De Visser et al. [31] studied the association of hypertension and type II diabetes in adult rats, comparing the Zucker Diabetic Fatty Rat (ZDF - a well stablished model of type II diabetes) and a genetic hybrid between the ZDF and the Spontaneously Hypertensive Heart Failure rat. The main finding for the diabetic rats was the presence of thinned myelin with a mild additive effect of hypertension that appeared only after 6 months of diabetes. Our results for the diabetic rats are somewhat similar to those from De Visser et al. [31] since we also demonstrated a small myelin area that was reflected on the total fiber size (smaller on WR + STZ compared to WR). On the other hand, on SHR animals, when compared to SHR + STZ, there was a tendency for increase in the average myelin area, associated with the increase in fiber size, suggestive of small myelinated fiber loss. In addition to these morphometric findings, SHR + STZ animals showed a great number of myelinated fibers with severe disruptions of the myelin sheath, particularly in large myelinated fibers (Figure 1).

Impairment of the motor conduction velocity in nerves from diabetic animals was described as related to large myelinated fibers dysfunction. Yamaguchi et al. [32] investigated the tail motor nerve conduction velocity in male Spontaneously Diabetic Torii-Lepr^fa^ rats (SDT Fatty). They demonstrated a reduction by 18% in the motor nerve conduction velocity in 24 weeks old SDT Fatty rats, compared to controls. In our study, the conduction velocity was not investigated because alterations in conduction velocity usually occur after a longer period then the experimental time we used (20 weeks of age, 12 weeks after diabetes induction). In our experimental model, because diabetes is severe (blood sugar levels sustained above 300 mg/dl without treatment), the animals do not survive under ethical conditions much longer than 12 weeks. Nevertheless, because larger fiber impairment is readily detectable in nerve conduction studies, and our study demonstrated that smaller fibers were more affected, we believe that the conduction studies in our experimental model would not add much.

It has been suggested that hyperglycemia is one factor for increasing Schwann cell metabolic stress. In fact, we recently have shown alterations of the Schwann cells mitochondria in nerves from acute STZ-diabetic rats [33]. The association between hypertension and hyperglycemia has also been suggested as a stress for the Schwann cells [9,31,34]. Our results showed that hypertension (without diabetes) significantly decreased the number and density of Schwann cells in the sural nerve compared to normotensive animals and, in the SHR + STZ, this number was reduced compared to SHR. The reduction in number of Schwann cells might be responsible, at least in part, for the severe myelin alterations we observed in SHR, with or without diabetes. Since the Schwann cells are the myelinating cells, an ideal relation or ratio between the number of the Schwann cells, the axon size and the myelin thickness is expected. The disruption of this ideal relation, like the reduction in the Schwann cells number can certainly cause myelin morphology alterations similar to the observed by us and others [9]. Metabolic stress in diabetic nerves was ascribed not only to hyperglycemia, but also to abnormal fatty acid metabolism, ischemic hypoxia and oxidative stress. The present study did not aim to investigate nerve metabolism but it has been shown that pharmacological interventions such as treatment with free radical scavenger can be effective in the prevention of the experimental diabetic neuropathy [35-37].

Thickening of the Schwann cell basement membrane is one of the alterations in diabetes, shown by ultrastrucutral investigations of peripheral nerves [14,33,38]. In our material it is evident that unmyelinated fibers are easily visible in diabetic animals (normotensive and hypertensive) compared to controls and also in SHR (Figure 1). An ultrastrucutral study of this material will help us to clarify this issue.

A reduction of myelinated nerve fiber size is the most characteristic and reproducible abnormality of peripheral nerve in experimentally induced diabetic rats [19,39-47]. This reduction is claimed to be due to a reduction in axon size caused by metabolic alterations in the axons [39,41,42,45,46], retarded growth rate [40,44], or increased hyperosmolarity [43]. More recently, the alterations of the endoneural microvasculature is also pointed out as a factor for the axon size reduction [12,13].

Our results of myelinated fiber alteration in hypertensive rats are very likely to be explained by the morphological changes in endoneural microvessels associated with hypertension, as described before [2,4]. Besides the larger number of collapsed blood vessels (with thickening of the walls and absence of lumen) and/or vessels with thick walls in SHR and SHR + STZ, a smaller fascicular area occupied by the capillary was statistically significant in these animals. Gregory et al. [9] observed reduction of endoneural blood vessels in SHR, associated to a decrease in sciatic nerve blood flow. Associated to these findings, they also showed reduced axonal diameter in SHR and diabetic SHR in the absence of changes to other structural parameters [9]. In line with our results, they affirmed that large fiber neuropathy in SHR resembled that of STZ-diabetic rats, but includes axons with thin myelin, suggestive of segmental demyelination/remyelination. The large fiber neuropathy present in our results for SHR can be identified by the reduced percentage of the large fibers peak on the distribution histograms (Figure 2), which is accentuated by the association between hypertension and diabetes. Adding to their results, we also show the small fiber neuropathy in SHR, through the myelinated fiber distribution shift to the right, also blunted by the association between hypertension and diabetes (Figure 2). The small fiber neuropathy in hypertension, similar to the one described in this study, was previously characterized in SHR though results obtained in our laboratory [2], by the decrease in number of myelinated fibers, the significant increase in myelinated fiber average size and the shift in fiber distribution histograms.

Experimental models of arterial infarct suggested that small nerve fibers are more vulnerable to ischemia than the large nerve fibers [48,49]. Thus, small fiber neuropathy in SHR can be associated to the damage of endoneural vessels [2,4]. In favor of the small fibers loss, an altered g ratio was also observed in the current study (Table 3 and Figure 2). The shift to the right observed in the g ratio distributions in SHR (diabetic and non-diabetic) could be suggestive of demyelination, as pointed out by Gregory et al. [9]. On the other hand, it is also described that larger fibers present larger g ratios compared to smaller fibers [50,51]. If the smaller fibers were lost, larger fibers will shift the g ratio distribution to the larger values.

The data presented here resulted from a comparison between SHR and Wistar rats, with or without experimentally induced diabetes. Spontaneously hypertensive rats (SHR) were first inbred from their normotensive counterparts, the Wistar-Kyoto rats (WKY). Thus, it would be expected for WKY to be used as the normotensive controls of SHR in diverse experimental protocols [7]. Nevertheless, Wistar rats (WR) have been recently used as the SHR controls as often as the WKY [52,53]. Peripheral nerve function is significantly affected by maturation and aging but rats described as adults have weights that vary in a range of about 180 g to 1000 g or more [17,54]. In the present study, animals with absolutely the same age but significantly different body weight presented similar fascicular area and diameter of the sural nerves. This indicates that the nerves developed in a similar way and the stress due to hypertension did not impact the overall SHR development. Actually, SHR are described as presenting smaller body weight compared to age matched WKY [9] as well as to Wistar rats [7].

## Conclusion

Our study gives morphological support to the existence of a neuropathy due to hypertension, which is among one of the most common risk factors for diabetic neuropathy. The association between the two neuropathies showed to be a complex alteration, involving and including both, large and small fibers neuropathy. Hypertension caused, indeed, an exacerbation of the alterations already observed in experimental models of diabetic neuropathy.
